# Molecular Mechanism of Stimulation of Na-K-ATPase by Leukotriene D4 in Intestinal Epithelial Cells

**DOI:** 10.3390/ijms22147569

**Published:** 2021-07-15

**Authors:** Niraj Nepal, Subha Arthur, Molly R. Butts, Soudamani Singh, Balasubramanian Palaniappan, Uma Sundaram

**Affiliations:** Department of Clinical and Translational Sciences, Appalachian Clinical and Translational Science Institute, Joan C. Edwards School of Medicine, Marshall University, Huntington, WV 25701, USA; nepal@marshall.edu (N.N.); arthursu@marshall.edu (S.A.); butts15@marshall.edu (M.R.B.); singhs@marshall.edu (S.S.); palaniappan@marshall.edu (B.P.)

**Keywords:** Na-K-ATPase, LTD4, crypt cells, intestinal epithelial cells

## Abstract

Na-K-ATPase provides a favorable transcellular Na gradient required for the functioning of Na-dependent nutrient transporters in intestinal epithelial cells. The primary metabolite for enterocytes is glutamine, which is absorbed via Na-glutamine co-transporter (SN2; SLC38A5) in intestinal crypt cells. SN2 activity is stimulated during chronic intestinal inflammation, at least in part, secondarily to the stimulation of Na-K-ATPase activity. Leukotriene D4 (LTD4) is known to be elevated in the mucosa during chronic enteritis, but the way in which it may regulate Na-K-ATPase is not known. In an in vitro model of rat intestinal epithelial cells (IEC-18), Na-K-ATPase activity was significantly stimulated by LTD4. As LTD4 mediates its action via Ca-dependent protein kinase C (PKC), Ca levels were measured and were found to be increased. Phorbol 12-myristate 13-acetate (PMA), an activator of PKC, also mediated stimulation of Na-K-ATPase like LTD4, while BAPTA-AM (Ca chelator) and calphostin-C (Cal-C; PKC inhibitor) prevented the stimulation of Na-K-ATPase activity. LTD4 caused a significant increase in mRNA and plasma membrane protein expression of Na-K-ATPase α1 and β1 subunits, which was prevented by calphostin-C. These data demonstrate that LTD4 stimulates Na-K-ATPase in intestinal crypt cells secondarily to the transcriptional increase of Na-K-ATPase α1 and β1 subunits, mediated via the Ca-activated PKC pathway.

## 1. Introduction

The primary function of the mammalian small intestine is the absorption of nutrients vital for the survival. The process of nutrient absorption occurs through a monolayer of epithelial cells (innermost layer of the mucosa), called enterocytes, present in the small intestine. These enterocytes are further divided into immature enterocytes (crypt cells) and mature enterocytes (villus cells). In the mammalian small intestine, the villus cells are generally considered to be absorptive thus possessing virtually all nutrient absorptive transport pathways. In contrast, crypt cells are typically considered to be secretory and are not known to have any nutrient absorptive pathways, except for Na-glutamine co-transport by SN2 (SLC38A5) in the brush border membrane (BBM).

The crypt cell BBM transporter SN2 requires a favorable Na-gradient, which is maintained by the Na-K-ATPase, an integral transmembrane protein located on the basolateral membrane (BLM) of these cells. In an animal model of chronic intestinal inflammation resembling human inflammatory bowel disease (IBD), it has been demonstrated that SN2 is stimulated in crypt cells, at least in part secondarily to the stimulation of Na-K-ATPase activity [1,2]. Moreover, it was postulated in this study that the stimulation of glutamine absorption mediated by SN2 in crypt cells might compensate for the decreased B0AT1 mediated glutamine absorption in the absorptive villus cells, to meet the overall nutritional demand of the enterocytes. However, it was observed that stimulation of glutamine absorption by the crypt cells in chronic intestinal inflammation does not recompense the loss of glutamine absorption by villus cells, thus demonstrating a net decrease in glutamine absorption by the enterocytes in chronic intestinal inflammation. Finally, it has also been shown that inhibition of the formation of leukotrienes in this animal model of IBD reverses the stimulation of Na-K-ATPase activity and subsequently SN2 activity in crypt cells [3]. However, it is unknown how LTD4 may stimulate Na-K-ATPase in crypt cells during chronic intestinal inflammation.

It is well established that various physiological and pathophysiological conditions alter the Na-K-ATPase activity through numerous mechanisms. It has been reported that there is a reduction of Na-K-ATPase activity in several diseases such as chronic neurodegenerative disorder [4], cardiovascular [5], and renal disease [6]. Similarly, malabsorption of a variety of nutrients in IBD is partly due to altered Na-K-ATPase activity [1,2,7,8,9]. During IBD, it has been shown that in villus cells, BBM Na-glucose co-transport (SGLT1), Na-alanine co-transport (ATB0), Na-bile acid co-transport (ASBT), and Na-glutamine co-transport (B0AT1) are all inhibited. All these co-transporters are altered at the level of the co-transporter in the BBM in the IBD intestine. At the cellular level, the downregulation of these co-transporters is due to the downregulation of BLM Na-K-ATPase leading to the loss of the intracellular Na^+^ gradient. However, it is interesting that only in crypt cells Na-K-ATPase activity was upregulated during chronic intestinal inflammation with concurrent stimulation of BBM Na-glutamine co-transporter SN2 [1], as SN2 is the only nutrient transport present in crypt cells and is critical for its health. Further, Na-K-ATPase activity and subsequently SN2 activity were reversed to normal levels by treatment with a corticosteroid, methylprednisolone [1,3,10]. This establishes that immune inflammatory mediators are responsible for altered BLM Na-K-ATPase activity as well as BBM SN2 activity in crypt cells during chronic intestinal inflammation. 

Arachidonic acid metabolites (AAMs) are prominent inflammatory mediators present in the mucosa of intestinal tissue from patients with IBD [11,12]. AAMs, specifically prostaglandin E2 (PGE2) and leukotriene D4 (LTD4) have been associated with the pathogenesis of several diseases including rheumatoid arthritis, asthma, psoriasis, multiple sclerosis, and IBD. It has been reported that LTD4 has also been associated with inflammatory processes [13] as well as intestinal secretion [14]. In various human cells and tissues, LTD4 through CysLT_1_R increase [Ca^2+^]_i_ and phosphatidylinositol (PI) metabolism activates MAPKs (Mitogen-activated protein kinases), induces cell proliferation and differentiation, actin reorganization, chemotactic migration, release of various inflammatory mediators, and regulation of hematopoietic stem cells mobilization [15]. LTD4 has also been involved in the stimulation of SN2 and Na-K-ATPase in IEC-18 cells [16]. However, the molecular mechanisms responsible for the stimulation of Na-K-ATPase by LTD4 in crypt cells during IBD have not been determined.

Na-K-ATPase is dynamically regulated to change its activity according to its physiological requirement. This holoenzyme is constituted of various subunits: alpha (α), beta (β), and gamma (γ) subunits. Of these subunits, two dissimilar subunits, α, and β, are required for the proper assemblage and function of the Na-K-ATPase [17,18]. However, the γ subunit is expressed in specific tissues and is optional for the functional activity of the Na-K-ATPase [19]. Of these subunits, the α subunit has the catalytic function: exchanging of 2K^+^ inside the cells to 3Na^+^ outside the cells as well as cleaving of ATP [20]. However, the β subunit facilitates maturation of the α subunit by the formation of α/β heterodimer and by transporting this enzyme to the plasma membrane [18]. Thus, both subunits are required for the efficient functioning of the Na-K-ATPase hence generating optimum sodium gradient inside the cell. This sodium gradient actuates secondary transport processes on BBM specifically Na-nutrient co-transporter, thus enabling intestinal epithelial cells to absorb nutrients efficiently. At least four isoforms of the α subunit (α1, α2, α3, and α4) and three isoforms of the β subunit (β1, β2, and β3) are reported to date. These isoforms are expressed in a tissue-specific manner [17,19,21]. The combination of different isoforms of α and β subunits to make up a series of Na-K-ATPase isoenzymes [22]. Each isoenzyme has different functional properties and is expressed differentially in a tissue- and cell-specific manner [23]. Of these isoforms, α1 and β1 are ubiquitously present in epithelial cells and are also present in the mucosa of the intestine. 

The Na-K-ATPase activity is tailored to change according to the physiological requirements of the cell. Several mechanisms regulate Na-K-ATPase activity—for example, the availability of the substrates (Na^+^, K^+^, and ATP). Additionally, the amount of the enzyme at the plasma membrane can be modified by changes in the rate of synthesis or degradation of the individual Na pump polypeptide and movement of the pump from the cytoplasm to the plasma membrane by exo/endocytotic vesicular transport [24]. Apart from these mechanisms, Na-K-ATPase activity at the cell surface is directly regulated by phosphorylation and dephosphorylation by protein kinases and protein phosphatases respectively [25,26,27]. Moreover, phosphorylation of transcription factors associated with Na-K-ATPase α and β subunits also changes the expression and activity of Na-K-ATPase [28,29].

Given this background, very little research has been conducted regarding the effect of LTD4 on the Na-K-ATPase in crypt cells, although the Na-K-ATPase is vital for the absorption of glutamine in crypt cells. Thus, this study aims to determine the molecular mechanism of stimulation of Na-K-ATPase activity by LTD4 in crypt cells during chronic intestinal inflammation.

## 2. Results

### 2.1. Stimulation of Na-K-ATPase Activity by LTD4

**Figure 1:**
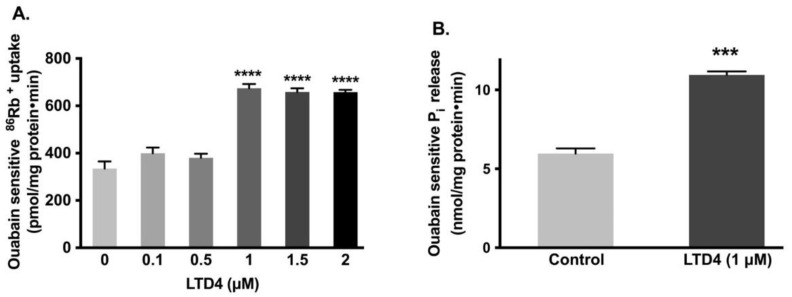
Effect of LTD4 (leukotriene D4) exposure for 24 h on Na-K-ATPase activity in IEC-18 cells. (**A**) Measurement of Na-K-ATPase activity by ^86^Rb^+^ uptake (*n* = 6, Dunnett’s multiple comparison test). (**B**) Measurement of Na-K-ATPase activity by P_i_ release in plasma membrane (*n* = 4, unpaired *t*-test). Values are represented as means ± SEM. *** *p* < 0.001, **** *p* < 0.0001 vs. 0 µM or control.

It has been shown that Na-K-ATPase activity in crypt cells, as well as LTD4 in the mucosa, is elevated in the chronically inflamed rabbit intestine [3]. IEC-18 cells grown to confluence (0-day post confluent) physiologically behave like crypt cells (e.g., express SN2 but not B0AT1) [30]. Therefore, to determine whether LTD4 is responsible for the stimulation of the Na-K-ATPase, IEC-18 cells were treated with various concentrations of LTD4, and the activity of the Na-K-ATPase was measured by uptake of the radioactive potassium analog, ^86^rubidium (^86^Rb^+^). Our observations showed that Na-K-ATPase was significantly stimulated by 1 µM of LTD4 (Figure 1A; 674.2 ± 45.19 picomole/mg protein/min) compared to control (334.8 ± 73.8). However, lower amounts of LTD4 did not show any stimulation (0.1 µM—399.4 ± 59.46, 0.5 µM—380.3 ± 42.93 picomole/mg protein/min) and amounts of LTD4 higher than 1 µM also did not show any additional stimulation of Na-K-ATPase. The stimulation of Na-K-ATPase activity in the plasma membrane by 1 µM of LTD4 was further corroborated with Na-K-ATPase assay P_i_ release (Figure 1B; 0 µM—5.96 ± 0.66, 1 µM—10.96 ± 0.45 nanomole/mg protein/min). 

### 2.2. Effect of LTD4 Exposure on Cell Viability

**Figure 2:**
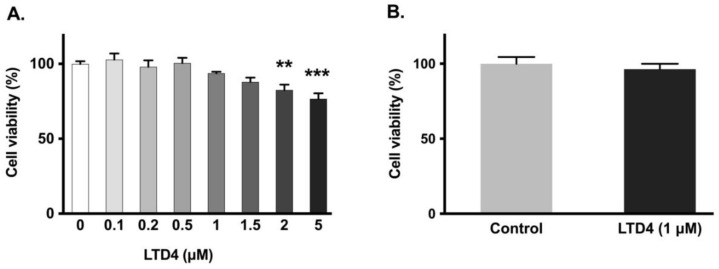
Effect of LTD4 exposure for 24 h on cell viability. (**A**) Measurement of cell viability by MTT assay (*n* = 6, Dunnett’s multiple comparison test). (**B**) Measurement of cell viability by trypan blue assay (*n* = 6, unpaired *t*-test). Values are represented as means ± SEM. ** *p* < 0.01, *** *p* < 0.001 vs. 0 µM.

To ensure that LTD4 did not negatively affect the viability of IEC-18 cells, MTT and trypan blue assays were performed. Cell viability was not significantly affected by LTD4 at or below 1.5 µM (Figure 2A), thus for all experiments, a concentration of 1 µM was subsequently used. This concentration was also validated with the trypan blue exclusion assay (Figure 2B). 

### 2.3. Leukotriene Receptor Antagonist Inhibited LTD4 Effect on Na-K-ATPase

**Figure 3:**
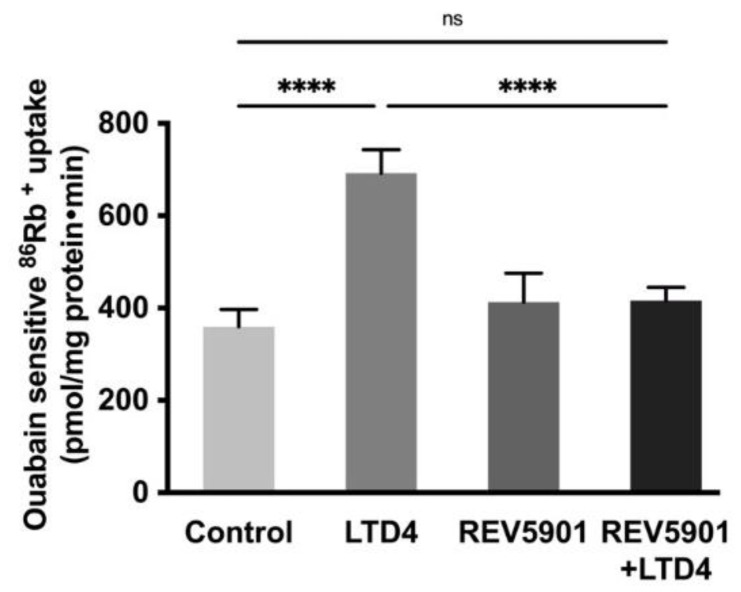
Effect of LTD4 receptor inhibitor REV5901 (5 µM) on Na-K-ATPase activity in IEC-18 cells after 24 h. Measurement of Na-K-ATPase activity by ^86^Rb^+^ uptake. Values are represented as means ± SEM (*n* = 6, Tukey’s multiple comparisons test). **** *p* < 0.0001; ns., not significant.

To determine whether the effect of LTD4 is mediated through its receptor, the effect of the LTD4 receptor antagonist, REV5901, was studied in IEC-18 cells. REV5901 is a competitive antagonist of peptido-leukotrienes and specifically blocks both cysteinyl leukotriene receptors 1 and 2, CysLT1 and CysLT2 [31]. It is known that IEC-18 cells have LTD4 receptors (CysLT1 and CysLT2) [32] and these receptors are inhibited by the specific LTD4 receptor inhibitor REV5901 (5 µM) [32]. After the pretreatment with REV5901, cells were treated with or without LTD4 for 24 h, followed by an ^86^Rb^+^ uptake assay. While LTD4 alone produced a significant increase in ^86^Rb^+^ uptake, neither REV5901 alone nor REV5901 + LTD4 affected ^86^Rb^+^ uptake compared to the control, as seen in Figure 3. These data indicate that the stimulatory effect of LTD4 is mediated through its receptor activation in IEC-18 cells. 

### 2.4. Role of Intracellular Calcium [Ca^2+^]_i_ on Na-K-ATPase during LTD4 Treatment

**Figure 4:**
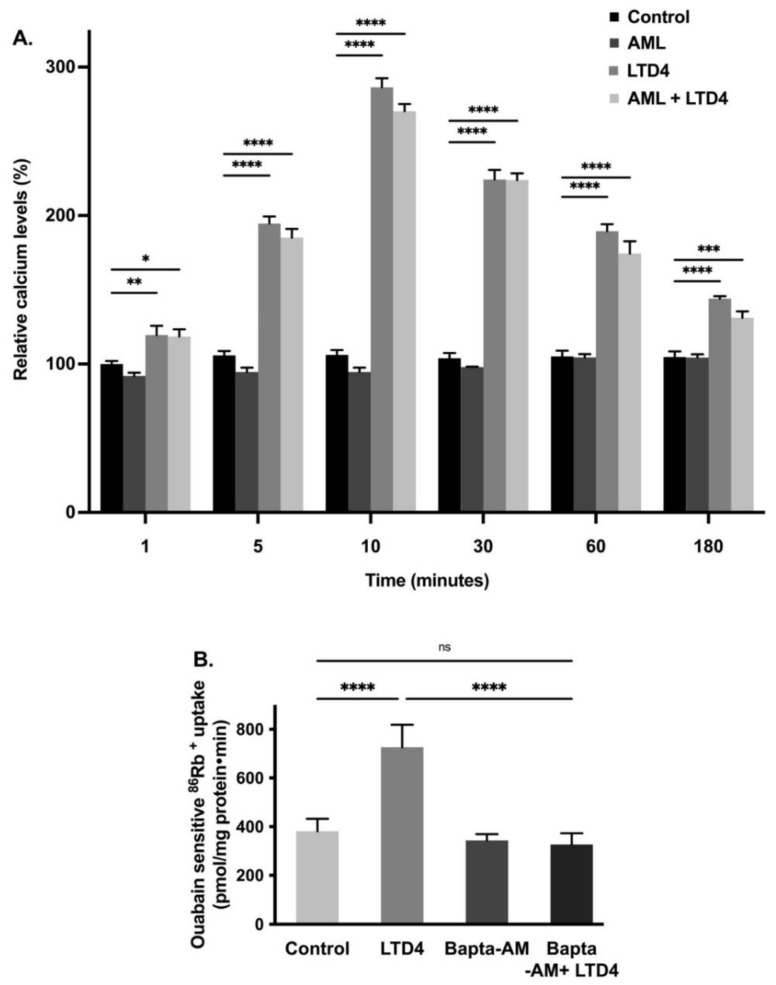
(**A**) Level of intracellular calcium at different time points during LTD4 treatment. Measurement of Ca^2+^ by Fluo-8 calcium flux assay. Values are relative to 1 min control (*n* = 8. Tukey’s multiple comparisons test). Control and amlodipine (AML, calcium channel blocker, 1 nM) alone did not change significantly at different time points. (**B**) Effect of calcium chelator BAPTA-AM (1 µM) on Na-K-ATPase activity in IEC-18 cells after 24 h. Measurement of Na-K-ATPase activity by ^86^Rb^+^ uptake (*n* = 6, Tukey’s multiple comparisons test). Values are represented as means ± SEM. * *p* < 0.05, ** *p* < 0.01, *** *p* < 0.001 vs. control and **** *p* < 0.0001 vs. control. ns., not significant.

Ca^2+^ is a second messenger and plays an essential role in signal transduction by either activation through ion channels or by G-protein coupled receptors [33]. This cation is vital for many physiological processes, including muscle contraction, neuronal excitability, cellular motility, cell growth, and apoptosis [34,35]. Therefore, we looked for the effect of LTD4 on [Ca^2+^]_i_ levels. Calcium channels were blocked with amlodipine (1 nM) in IEC-18 cells and then the cells were treated with LTD4. The [Ca^2+^]_i_ levels increased in cells treated with LTD4 as shown in Figure 4A. Intracellular Ca^2+^ increased almost 2-fold within 5 min and peaked at 10 min (~3-fold) with LTD4. Levels reduced gradually at later time points (30 min: ~2.3-fold, 60 min: ~1.8-fold, 180 min: ~1.3-fold) but were still significantly higher than control. 

To determine whether elevated intracellular Ca^2+^ is responsible for the stimulation of the Na-K-ATPase, we used the calcium chelator BAPTA-AM. IEC-18 cells were pretreated with 1,2-bis(o-aminophenoxy) ethane-N,N,N’N’-tetraacetic acid (BAPTA-AM-1 µM) followed by measuring ^86^Rb^+^ uptake. The Na-K-ATPase activity was found to be stimulated with LTD4 whereas BAPTA-AM treatment inhibited LTD4-mediated stimulation of the Na-K-ATPase activity, as shown in Figure 4B (control-381.6 ± 50.54, LTD4-726.3 ± 92.26, BAPTA-AM-344 ± 25.6, BAPTA-AM + LTD4-326.5 ± 46.8 picomole/mg protein/min). These data indicate that LTD4 increases intracellular calcium, which leads to stimulation of the Na-K-ATPase activity.

### 2.5. Effect of Inhibition and Activation of the PKC Pathway on Na-K-ATPase Activity in IEC-18 Cells

**Figure 5:**
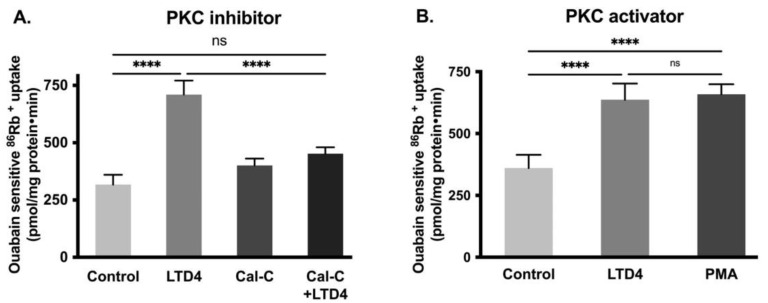
Effect of Protein kinase C (PKC) inhibitor and activator on Na-K-ATPase activity in IEC-18 cells after 24 h. (**A**) PKC inhibitor calphostin-C (0.1 µM). (**B**) PKC activator PMA (1 µM). Measurement of Na-K-ATPase activity by ^86^Rb+ uptake. Values are represented as means ± SEM (*n* = 6, Tukey’s multiple comparisons test). **** *p* < 0.0001 vs. control; ns., not significant.

Previous studies have shown that PKC phosphorylates Na-K-ATPase subunits, which subsequently affects the activity of the Na-K-ATPase [36]. To see whether PKC mediates the LTD4 effect, cells were pretreated with the PKC inhibitor calphostin-C (0.1 μM) for an hour. After pretreatment, cells were treated with LTD4 for 24 h, followed by ^86^Rb^+^ uptake. Pretreatment with calphostin-C inhibited the LTD4-mediated stimulation of Na-K-ATPase activity (Figure 5A; control—317.3 ± 42.8, LTD4—710.3 ± 61.17, Cal-C—400.8 ± 30.14, Cal-C+ LTD4—452 ± 28.31 picomole/mg protein/min). Similarly, the PKC activator PMA (1 μM) also stimulated Na-K-ATPase comparable to LTD4 (Figure 5B; control—360.9 ± 53.12, LTD4—636.9 ± 65.51, PMA—659.3 ± 39.74). These data indicate that PKC mediates LTD4 stimulation of Na-K-ATPase in IEC-18 cells.

### 2.6. Na-K-ATPase α1 and β1 Subunit mRNA Abundance during LTD4 Treatment

**Figure 6:**
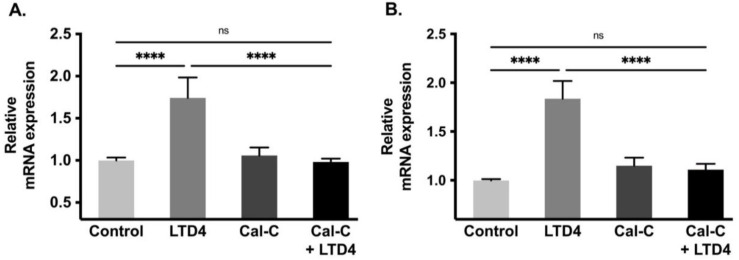
Quantification of Na-K-ATPase α1 and Na-K-ATPase β1 mRNA through quantitative real-time PCR (qRT-PCR) when treated with LTD4 and calphostin-C (PKC inhibitor) for 24 h. Values are relative to control and normalized to β-actin. (**A**) Na-K-ATPase α1. (**B**) Na-K-ATPase β1. Values are represented as mean ± SEM (*n* = 6, Tukey’s multiple comparisons test). **** *p* < 0.0001; ns., not significant.

Na-K-ATPase functional activity is primarily due to its α subunit whereas the β subunit does not have pumping activity but provides proper transportation of the α subunit to the plasma membrane making it fully functional. Therefore, to determine whether the change in Na-K-ATPase activity is transcriptionally regulated, we performed a qRT-PCR analysis. There was a significant increase in mRNA level of both Na-K-ATPase α1 and β1 subunits when exposed with LTD4, by 1.6-fold and 1.8-fold respectively (Figure 6A,B). However, the Na-K-ATPase α1 and β1 subunit mRNA levels remained unaltered compared to control when cells were treated with Cal-C+ LTD4. These data indicate that Na-K-ATPase α1 and β1 subunits are transcriptionally upregulated when treated with LTD4 and that PKC plays an important role in LTD4-mediated regulation of Na-K-ATPase. 

### 2.7. Na-K-ATPase α1 and β1 Subunit Protein Expression during LTD4 Treatment

**Figure 7:**
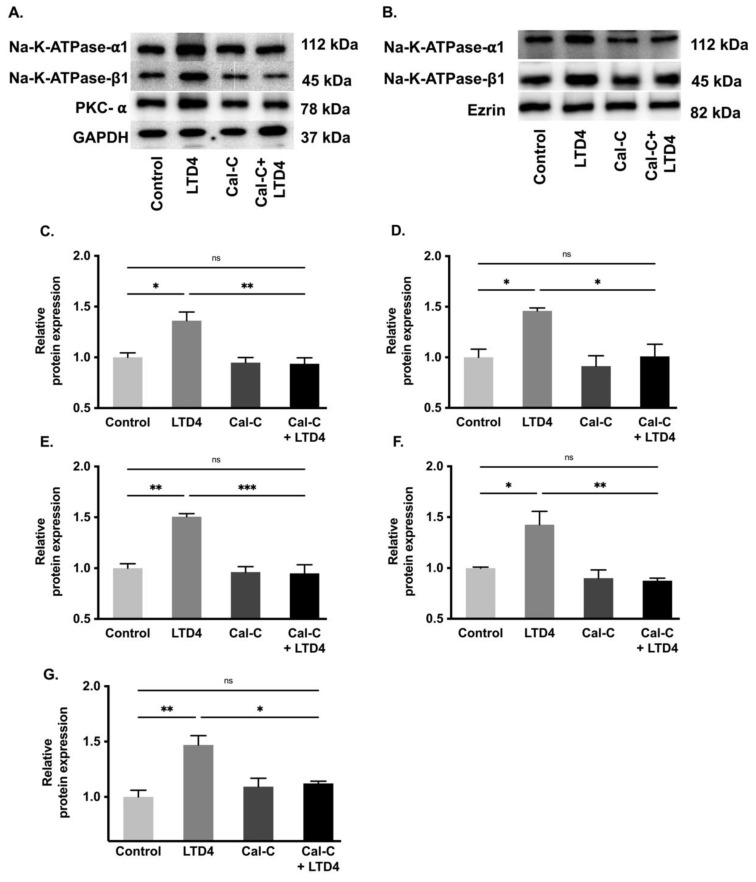
Quantification of Na-K-ATPase α1 and Na-K-ATPase β1 through Western blots when treated with LTD4 and calphostin-C (PKC inhibitor) for 24 h. (**A**) Representative Western blots of Na-K-ATPase α1, Na-K-ATPase β1, PKC-α and GAPDH (loading control) in whole cell lysate (WCL). (**B**) Representative Western blots of Na-K-ATPase α1, Na-K-ATPase β1, and Ezrin (loading control) in plasma membrane (PM). Densitometric quantitation of blots, (**C**) Na-K-ATPase-α1, (**D**) Na-K-ATPase-β1, (**E**) PKCα in WCL and (**F**) Na-K-ATPase-α1, (**G**) Na-K-ATPase-β1 in PM. Values are relative to control and normalized to GAPDH or Ezrin. Values are represented ± SEM, (*n* = 4, Tukey’s multiple comparisons test). * *p* < 0.05, ** *p* < 0.01; *** *p* < 0.001, ns., not significant.

**Figure 8:**
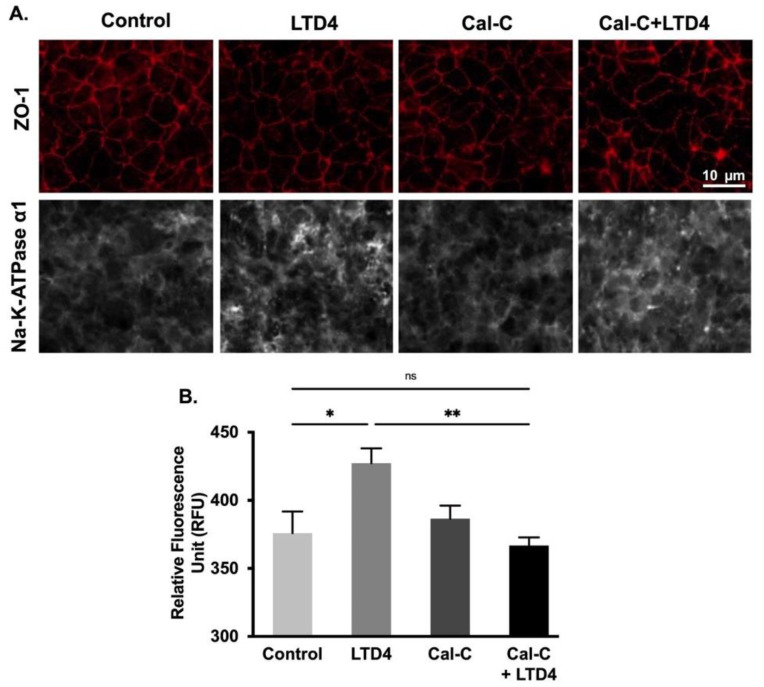
Quantification of Na-K-ATPase α1 through immunocytochemistry when treated with LTD4 and calphostin-C (PKC inhibitor) for 24 h. (**A**) Representative images of Na-K-ATPase α1 (gray), and ZO-1 (Red), (20×). (**B**) Quantification of Na-K-ATPase α1 (green channel). Values are represented as mean ± SEM, (*n* = 8, Tukey’s multiple comparisons test). *, *p* < 0.05, **, *p* < 0.01; ns., not significant.

To determine whether changes in Na-K-ATPase activity are due to altered protein expression of the Na-K-ATPase α1 and β1 subunits, Western blots analysis of whole cell lysate and plasma membrane (Figure 7) and immunocytochemistry of IEC-18 cells (Figure 8) were performed. Densitometric analysis of Western blots revealed that the level of Na-K-ATPase α1 protein expression was significantly increased by LTD4 treatment, whereas the LTD4 effect was inhibited when pretreated with Cal-C (Figure 7A–C,F) both in whole cell lysate and plasma membrane. Immunocytochemistry performed on IEC-18 cells also showed that there was a significant increase in Na-K-ATPase α1 while treated with LTD4 and the effect was nullified by Cal-C (Figure 8A,B). Similarly, there was also a significant increase in Na-K-ATPase β1 subunit protein expression in the whole cell lysate and plasma membrane when treated with LTD4, and this level reverted to normal when treated with Cal-C (Figure 7A,B,D,G). Therefore, the mRNA expression of Na-K-ATPase α1 and Na-K-ATPase β1 subunits correlated with its protein expression in the whole cell lysate and plasma membrane. Taken together, these findings show that LTD4 regulates Na-K-ATPase transcriptionally by increasing mRNA and protein levels of the α1 and β1 subunits through the PKC pathway. 

## 3. Discussion

In the mucosa of the chronically inflamed intestine, LTD4 levels are significantly elevated. More importantly, LTD4 has been shown to alter nutrient absorption in the villus as well as the crypt cells in the chronically inflamed intestine through unique pathways and molecular mechanisms [16,32,37,38,39]. LTD4 been shown to inhibit alanine transport in the villus cells by affecting the affinity of the alanine transporter ASCT1 through the PKC pathway [37]. In contrast, during inflammation, LTD4 has been shown to stimulate glutamine absorption, mediated through SN2, the sole nutrient absorptive mechanism present in the crypt cells [16]. In both of these instances, the alteration in the activities of these specific nutrient transporters was secondary to altered Na-K-ATPase activities: ASCT1 inhibition was secondary to Na-K-ATPase inhibition in the villus cells [37] and SN2 stimulation was secondary to Na-K-ATPase stimulation in crypt cells [16]. Though the regulation of these nutrient transporters by LTD4 has been well understood, it is not clear if LTD4 is directly responsible for altered Na-K-ATPase activities in intestinal epithelial cells during inflammation. We hypothesized that LTD4 may stimulate Na-K-ATPase activity to facilitate the stimulation of SN2 activity in crypt cells during chronic intestinal inflammation. This study for the first time examines the effect of LTD4 on Na-K-ATPase activity and provides the mechanism of stimulation of the Na-K-ATPase by LTD4 in the intestinal crypt cells during chronic intestinal inflammation. 

To explore the mechanism of regulation of Na-K-ATPase activity by LTD4 in the crypt cells, rat intestinal epithelial cells (IEC-18) were used. These cells at 0-day post-confluence phenotypically resemble intestinal crypt cells and mature into villus-like cells by day 4 post-confluence [30]. Comparable to native crypt cells, IEC-18 cells at day 0 have SN2 on the BBM but not any other nutrient absorptive processes, and have Na-K-ATPase, located on the BLM, to provide the favorable Na gradient for SN2 [16]. Thus, these cells are an ideal in vitro model of intestinal crypt cells. 

This study demonstrated that 1 μM LTD4 for 24 h is both non-toxic and optimal for stimulation of Na-K-ATPase activity in IEC-18 cells. This stimulation of Na-K-ATPase activity is through LTD4 receptor activation. At the level of the intracellular signaling, receptors CysLT1 and CystLT2 mediate their action through various G-proteins that subsequently alter intracellular Ca^2+^ or cAMP [38,40,41,42,43]. This study demonstrated that LTD4 increased intracellular Ca^2+^ when exposed to LTD4; thus, when Ca was chelated, the reversal in the stimulation of Na-K-ATPase activity was observed. Therefore, this indicates that stimulation of Na-K-ATPase activity is mediated through increased [Ca^2+^]_i_. 

Alterations in intestinal epithelial cell absorption and secretion are mediated by immune inflammatory mediators, known to be released in the chronically inflamed intestine [1,2,7,8]. The alteration in absorptive mechanisms during intestinal inflammation may be due to many immune inflammatory mediators including prostaglandins, leukotrienes (LTs), chemokines, interleukins, and/or reactive nitrogen and oxygen species [44]. Indeed, many of these inflammatory mediators have been shown to regulate nutrient and electrolyte transport processes during chronic intestinal inflammation [39,45,46,47,48]. Of the arachidonic acid metabolites known to be increased in the chronically inflamed intestinal mucosa, there is a wealth of evidence that LTs (LTB_4_, LTC_4_, LTD4, and LTE_4_) are involved in the alteration of intestinal electrolyte transport in IBD patients [49]. Other studies have shown that the LOX pathway is involved in the stimulation of Na-glutamine transporter (SN2) and Na-K-ATPase activity in crypt cells in the inflamed intestine of rabbits [3]. Hence, it was reasonable to postulate that LTD4 mediates the stimulation of Na-K-ATPase activity in crypt cells and identifies the specific mechanisms by which LTD4 stimulates the Na-K-ATPase. 

The LTD4-mediated increase of [Ca^2+^]_i_ may in-turn activate Ca^2+^ dependent kinases and downstream signaling [38,40,41,42,43]. Based on the external stimulus, PKC is involved in the reduction or activation of Na-K-ATPase activity, depending upon species and tissues studied [50,51,52]. Moreover, the stimulation of various isoforms of PKC can have a differential effect on the Na-K-ATPase. Conventional PKC α and β are associated with decreased activity of Na-K-ATPase by endocytosis [53,54], whereas other isoforms of PKC (ε and δ) have been shown to stimulate Na-K-ATPase activity through the ERK1/2 pathway [36,55]. In the present study, the PKC pathway was activated by LTD4. When calphostin-C inhibited PKC, LTD4-mediated stimulation of Na-K-ATPase activity was attenuated, thus indicating that Ca^2+^-activated PKC is responsible for the stimulation of Na-K-ATPase activity. Future studies will need to identify the exact PKC isoform responsible for the LTD4-mediated stimulation of Na-K-ATPase actiivity. 

Upon activation of PKC, the modulation of the Na-K-ATPase might be due to (1) direct phosphorylation of Na-K-ATPase α and γ subunits, or (2) alteration in transcription factors associated with various subunits of the Na-K-ATPase [29]. In the present study, LTD4 increased the mRNA levels for the α1 and β1 subunits of the Na-K-ATPase and subsequently the protein levels of these subunits in the plasma membrane. Transcription factors such as specific protein (SP); Sp1, Sp2, and Sp3 are ubiquitously expressed in various tissue whereas Sp4 is mainly confined to neurons and testis [29]. Among these, Sp1 and Sp3 were found to increase the transcription of the α1 and β1subunitsts by binding to the promoter region of these subunits [56,57]. Similarly, phosphorylation of transcription factor CREB induces the increased α1 mRNA level without altering the β1 subunit mRNA level [58]. Additionally, the binding of ZEB1 (AREB6) to the promoter region of the α1 subunit increases the protein levels of the α1 subunit in skeletal muscle [36]. Future studies will decipher the potential transcription factor via which LTD4 may stimulate Na-K-ATPase activity in crypt cells.

## 4. Materials and Methods

### 4.1. Reagents

All reagents used in the experiments were purchased from Cayman Chemicals (Ann Arbor, MI, USA): leukotriene D4 (LTD4) (Cat# 20310), REV 5901 (Cat# 70600), calphostin-C (Cat# 15383), REV 5901 (Cat# 70600), phorbol 12-myristate 13-acetate (Cat# 10008014), BAPTA-AM (Cat# 15551), Amlodipine (Cat#14838). All reagents were dissolved in DMSO except LTD4, which was dissolved in ethanol to make the stock solutions. The final working concentration of the solutions contained less than 0.5% (*v*/*v*) of DMSO or ethanol. The toxicity of all the drugs was assessed, and the safest dose was used for further experiments (Figure S1). 

### 4.2. Cell Culture

The rat small intestine cell line IEC-18 (American Type Culture Collection), between passages 5 and 20, was used for all the experiments. Cells were maintained in Dulbecco’s modified Eagle’s medium (DMEM), supplemented with 10% (*v*/*v*) fetal bovine serum, 100 U/L human insulin, 0.25 mM β-hydroxybutyric acid, and 100 units/mL penicillin and streptomycin. These cells were cultured in a humidified atmosphere of 10% CO_2_ at 37 °C. Cells were fed with fresh DMEM every other day. When the cells reached 100% confluence, it was considered as 0 days and the cells exhibit crypt like functions [30].

### 4.3. Cell Viability Assays

For assessing the cell viability, the MTT (3-(4,5-dimethylthaizol-2-yl)-2,5-diphenlytetrazolium bromide) assay was performed using Vybrant MTT cell proliferation kit (ThermoFisher Scientific, Cat# V-13154, Waltham, MA, USA). Cells (5 × 10^4^ cells) per well were seeded in 96-well plates and cultured until confluent, then treated with test chemicals for desired time. After the desired time, media was aspirated and replaced with 100 μL of fresh medium. Then MTT solution (12 mM; 10 μL in PBS) was added and incubated at 37 °C for 2 h. The media was replaced with 100 μL of lysis buffer (SDS-HCl) solution to each well and incubated for 12 h at 37 °C. The solution was mixed thoroughly before taking an absorbance on the plate reader (Spectramax i3x, Molecular Devices, San Jose, CA, USA) at 570 nm. Trypan blue exclusion assay was also performed to measure the cell viability using 0.4% Trypan blue (ThermoFisher Scientific, Cat# 15250061, Waltham, MA, USA). Cells were plated in 24-well plates and cultured until confluent, then treated with or without LTD4 for 24 h. After 24 h, cells were washed 2× with PBS, and cells were dissociated using 0.25% trypsin. The dissociated cells were mixed in 1:1 proportion with trypan blue and counted under a compound-light microscope using a hemocytometer. 

### 4.4. Crude Plasma Membrane Preparation

Plasma membrane (crude) was isolated from cells according to the method of Havrankova et al. [59]. Subsequently, cells were mixed with a 2.5-fold volume of 0.001 M NaHCO_3_ (pH 7.4) and homogenized 3× (10 s each) with a homogenizer (IKA Works Inc., Cat# 823707, T25 S, Wilmington, NC, USA). These homogenized cells were centrifuged at 600× *g* for 30 min. The resultant supernatant was centrifuged for 30 min at 20,000× *g*. The membrane was washed twice with 0.001 M NaHCO_3_. The final pellet was resuspended in 0.04 M Tris-HCl buffer (pH 7.4) containing 0.1% BSA. All procedures were carried out at 4 °C. 

### 4.5. Na-K-ATPase Activity Assay

The Na-K-ATPase activity was measured as Pi liberated [60] in plasma membrane fractions from cells according to the protocol of Forbush et al. [60]. Briefly, a solution I (Tris HCL (pH 7.4), MgSO_4_ (0.1 M), KCl (0.1 M), NaCl (0.1 M)) was first prepared with or without ouabain (Na-K-ATPase inhibitor). Then 20 µg of plasma membrane preparation was added to the solution I and incubated for 5 min at 37 °C. Subsequently, adenosine triphosphate (ATP; 2 mM) was added to solution I with the plasma membrane preparation and incubated for another 15 min at 37 °C. Then a solution II (ascorbic acid 0.49 M, 1 N HCl, 20% SDS, 10% ammonium molybdate) was added, followed by incubation for another 10 min in the ice water bath. The reaction was stopped by the addition of solution III (2% arsenite, 2% sodium citrate, 2% acetic acid) followed by incubation for 10 min at 37 °C. Finally, the solution was read at 705 nm in a spectrophotometer. The enzyme-specific activity was expressed as nanomoles of Pi released per milligram protein per minute. 

### 4.6. RNA Isolation and qRT-PCR

RNA was isolated from various groups using the RNeasy Mini Kit (Qiagen, Cat# 74104, Germantown, MD, USA). Once RNA was isolated, cDNA was synthesized from total RNA using SuperScript III (ThermoFisher Scientific, Cat# 12574026; Waltham, MA, USA). The cDNA was synthesized using random hexamers provided in the kit. An equal amount of cDNA was used as a template to perform real-time quantitative PCR (qRT-PCR) using TaqMan^TM^ Universal PCR master mix (ThermoFisher Scientific, Cat# 4304437; Waltham, MA, USA) according to the manufacturer’s protocol. Rat Na-K-ATPase α1 (Taqman Gene Expression Assay, Thermofisher, Assay number Rn01533986_m1) and β1-specific (Taqman Gene Expression Assay, Thermofisher, Assay number Rn00565405_m1) primers were used for the qRT-PCR studies and β-actin (Taqman Gene Expression Assay, Thermofisher, Assay number Rn00667869_m1) was used as a housekeeping gene to normalize the expression of samples.

### 4.7. 86 Rb^+^ Uptake for Na-K-ATPase Activity

IEC-18 cells were grown and maintained as mentioned above. Uptake studies were performed in cells grown on 24-well transwell inserts (Millipore Sigma, Cat# CLS3396, pore size 0.4 μ, St. Louis, MO, USA). 5 × 10^4^ cells were seeded IEC-18 cells were seeded in each transwell. Upon confluency, Na-K-ATPase activity studies were performed using radioactive Rubidium (^86^Rb^+^, PerkinElmer, Akron, OH, USA). On the day of uptake, cells were washed with serum-free DMEM (SFM) once and incubated for another 1 h with SFM. Then cells were incubated for another 10 min at 37 °C in SFM containing 20 μM monensin on both sides of the transwell. Subsequently, cells were washed with SFM and ^86^Rb^+^ uptake was performed by incubating cells for 15 min with a reaction mixture containing SFM and ^86^Rb^+^ (~1 μCi/well) on the basal side of the membrane in the presence and absence of ouabain (1 mM). The reaction was stopped by the addition of stop buffer (ice-cold MgCl_2_) and subsequently washed three times with stopping buffer. Finally, the cells were lysed with 800 μL of 1 N NaOH, incubation for 30 min at 70 °C, and then mixed with 4 mL of Ecoscint A (National diagnostics, Cat# LS-273). The vials were kept overnight and radioactivity per well was determined in a Beckman Coulter 6500 scintillation counter. 

### 4.8. Calcium Measurement

Calcium measurement was performed using the Fluo-8 calcium flux assay kit (Abcam, Cat# ab112129). Cells were seeded with an equal number of cells in 96-well plates and cultured until confluent, then treated with test chemicals for desired time. Subsequently, the supernatant was removed and replaced with 100 μL Fluo-8 dye-loading solution per well and incubated for 30 min at room temperature. Next, the fluorescence intensity was measured at Ex/Em of 490/525 nm using a fluorescence plate reader (Spectramax i3x, Molecular Devices, San Jose, CA, USA).

### 4.9. Immunocytochemistry (ICC) Staining

IEC-18 cells were grown on coverslips until confluent. Different groups of cells were treated as mentioned before. Following the treatment for 24 h, cells were fixed with 100% methanol (chilled at −20 °C) at room temperature for 5 min and subsequently permeabilized with PBST (PBS + 0.5% Tween 20) for 10 min. Then cells were blocked with 3% BSA in PBST for 30 min and incubated for an hour at room temperature with primary antibody ZO-1(anti-rabbit; ThermoFisher Scientific, Cat# 40-2200, Waltham, MA, USA), and Na-K-ATPase α1(anti-mouse; Millipore Sigma, Cat# 05-369, St. Louis, MO, USA). The secondary antibody (Alexa Fluor 488; ThermoFisher Scientific, Waltham, MA, USA) was added, and the cells were incubated at 37 °C for another 1h. Cells were mounted with DAPI mounting medium (Abcam, Cat# ab104139, Cambridge, MA, USA) and sealed with nail polish to prevent cells from drying. An EVOS microscope (ThermoFisher Scientific, Waltham, MA, USA) was used to capture images with constant exposure time for all experiments. ImageJ was used for analysis. 

### 4.10. Western Blot Analysis

Plasma membrane proteins was prepared from plasma membrane fractions prepared from different samples as described above. Protein was quantified and an equal amount of protein per group was taken for denaturation. Then the protein was denatured using the 1:1 volume of 2× Laemmli sample buffer (Bio-Rad, Cat# 1610737, Hercules, CA, USA) and separated by electrophoresis (100 Volt for 1.5 h) on an 8% polyacrylamide gel. Subsequently, the proteins on the gel were transferred to a polyvinylidene fluoride membrane (PVDF) for another 1.5 h (4 °C). The membrane was blocked with 5% milk or BSA in TBS (20 mM Tris pH 7.5, 150 mM NaCl) with 0.1% Tween-20 and then incubated with primary antibody against Na-K-ATPase α1 (Anti-mouse; Millipore Sigma, Cat# 05-369, St. Louis, MO, USA) or Na-K-ATPase β1 (Abcam, Cat# ab2873, Cambridge, MA, USA) overnight at 4 °C. Then the membranes were washed three times each with TBS and TBST, followed by incubation with secondary antibody for 1 h. Again, the membranes were washed three times each with TBS and TBST. An ECL western blotting detection reagent (GE Healthcare Biosciences, Piscataway, NJ, USA) was used to detect the immobilized protein. The chemiluminescence was detected using the FluorChemM instrument (Alpha Innotech, San Leandro, CA, USA) and analyzed with its software. Ezrin antibody (Abcam, Cat# ab4069, Cambridge, MA, USA) and GAPDH (anti-mouse; ThermoFisher Scientific, Cat# MA-15378, Waltham, MA, USA) was used to normalize the expression levels of proteins in the plasma membrane and whole cell lysate respectively. 

### 4.11. Protein Determination

The Bio-Rad DC Protein Assay Kit (Hercules, CA, USA) was used to measure the protein concentration. Various concentrations of BSA (0–2 mg/mL) were used as standards. The 10 µL sample was mixed with 25 µL of DC Protein Assay reagent A (Bio-Rad, Cat# 500-0113, Hercules, CA, USA) and incubated for 2 min in 96-well plate. Subsequently, the sample was incubated for another 15 min after the addition of 200 µL DC Protein Assay reagent B (Bio-Rad, Cat# 500-0114, Hercules, CA, USA). Finally, the sample was read at OD (Optical density) 750 nm using a plate reader (Spectramax i3x, Molecular Devices, San Jose, CA, USA).

### 4.12. Statistical Analysis

All groups presented have at least *n* = 4 per group, repeated with different passages. The values are presented as mean ± SEM, and *p*-values of <0.05 were taken to indicate statistical significance. All the data were analyzed using a unpaired *t*-test or two-way analysis of variance (ANOVA). A two-way analysis of variance, followed by Tukey’s and Dunnett’s multiple comparisons post hoc test was used for testing the differences among the groups. Statistical significance is indicated as * *p* < 0.05, ** *p* < 0.01, *** *p* < 0.001 vs. control and **** *p* < 0.0001. GraphPad Prism software (Version 9.1.0, San Diego, CA, USA) was used for statistical analysis.

## 5. Conclusions

**Figure 9:**
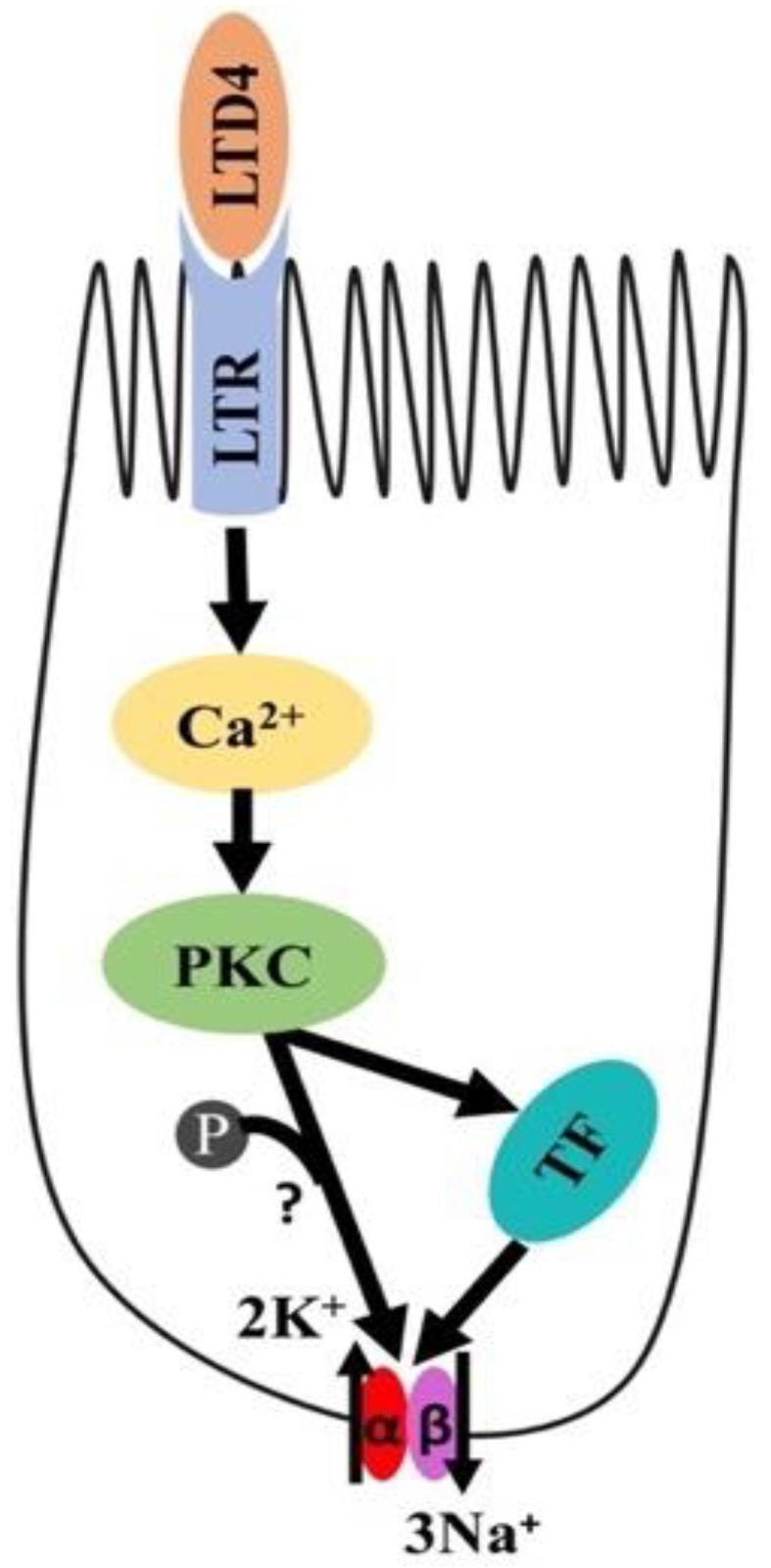
Proposed mechanism of LTD4-mediated regulation of Na-K-ATPase in intestinal epithelial cells. LTD4—leukotriene D4, LTR—leukotriene receptor, PKC—protein kinase C, TF—transcription factor.

In conclusion, as shown in Figure 9, LTD4-mediated stimulation of Na-K-ATPase activity in intestinal crypt cells is due to the activation of Ca-dependent PKC, which in turn activates different transcription factors regulating the expression of α1 and β1 subunits of the Na-K-ATPase. Thus, in the chronically inflamed intestine, LTD4-mediated the stimulation of crypt cell Na-K-ATPase activity via PKC and the enhanced transcription of α1 and β1 subunits of the Na-K-ATPase.

## Data Availability

Not applicable.

## Supplementary Materials

The following are available online at https://www.mdpi.com/article/10.3390/ijms22147569/s1.
