# Supplementary material for: Molecular Mechanism of Stimulation of Na-K-ATPase by Leukotriene D4 in Intestinal Epithelial Cells
Source: Int J Mol Sci. 2021 Jul 15;22(14):7569. doi: 10.3390/ijms22147569 (PMC8303499; doi:10.3390/ijms22147569)
Supplement: Supplementary file 1 [file ijms-22-07569-s001.zip › ijms-1215659-supplementary.pdf]

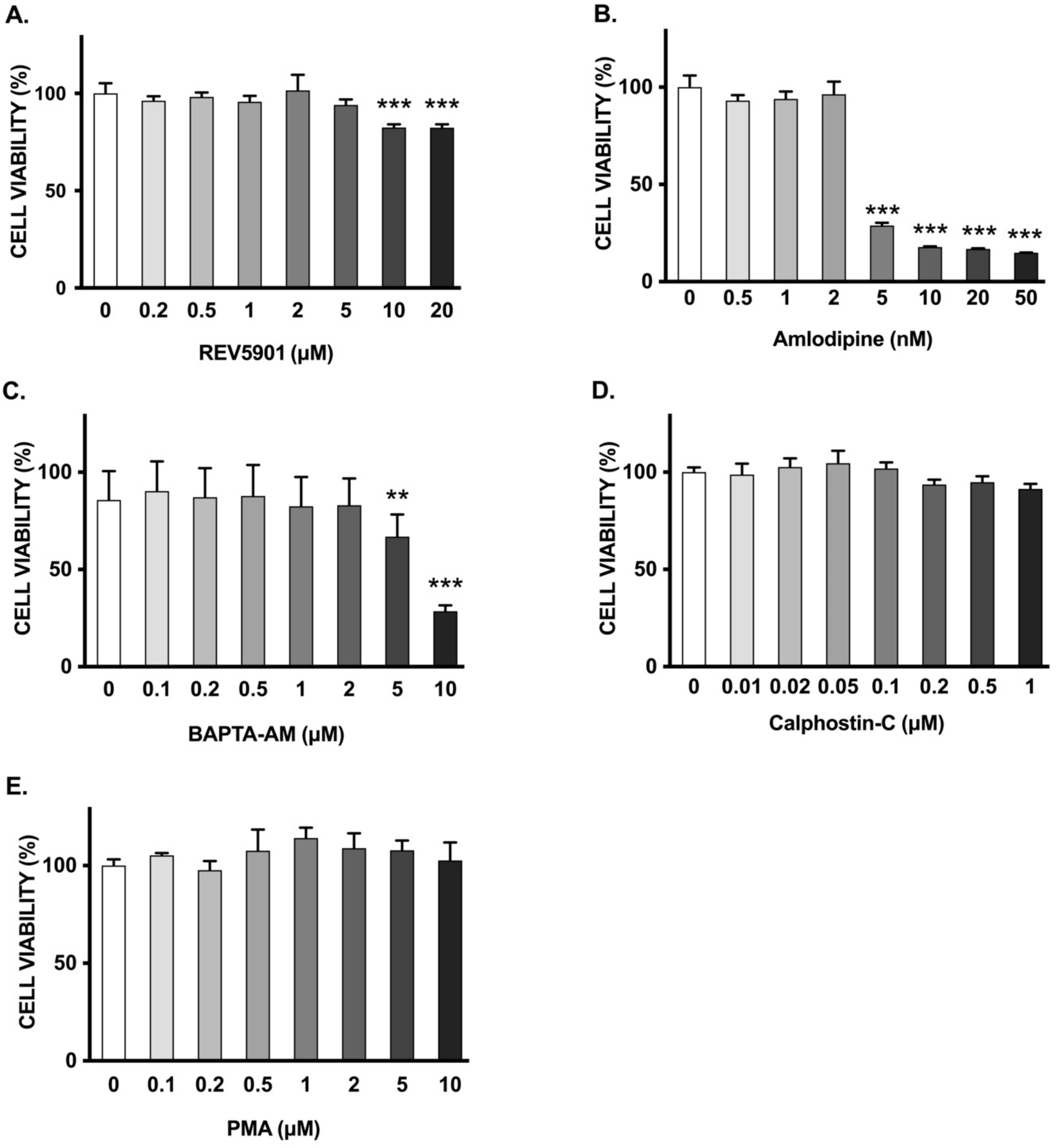

Figure S1. Effect of different concentration of reagents (drugs) exposure for 24 hours on Intestinal epithelial cell (IEC-18) viability. A. REV5901. B. Amlodipine. C. BAPTA-AM. D. Calphostin-C. E. PMA (Phorbol 12-myristate 13-acetate). Measurement of cell Viability by MTT assay. Values are relative to 0 µM and are represented as means ± SEM (n=6, *Dunnetts's multiple comparisions test*). **, $p < 0.01$ vs 0 µM, ***, $p < 0.001$ vs 0 µM.
